# Antiepileptic Drug Adherence and Its Associated Factors among Epilepsy Patients on Follow-ups at Amanuel Mental Specialized Hospital, Ethiopia

**DOI:** 10.4314/ejhs.v32i5.6

**Published:** 2022-09

**Authors:** Shegaye Shumet, Mesele Wondie, Getinet Ayano, Henock Asfaw, Tilahun Kassew, Gebremeskel Mesafint

**Affiliations:** 1 Department of psychiatry, University of Gondar, Gondar, Ethiopia; 2 Amanuel Mental Specialized Hospital, Addis Abeba, Ethiopia; 3 School of nursing and midwifery, college of health and medical science, Haramaya University; 4 Department of Psychiatry, College of health science, Mizan-Tapi University, Mizan-Aman, Ethiopia

**Keywords:** Antiepileptic medication, Adherence, Epilepsy

## Abstract

**Background:**

Medication adherence is a fundamental determinant of effective treatment. However, people with epilepsy have poor compliance with their treatment because of the chronic nature of the disease. Limited studies have been conducted to address antiepileptic medication adherence in Africa, including Ethiopia. Thus, the aim of this study was to assess antiepileptic drug adherence and its asociated factors among patients with epilepsy attending outpatient department of Amanuel Mental Specialized Hospital.

**Methods:**

A cross-sectional study design was conducted on 439 patients with epilepsy in Amanuel Mental Specialized Hospital. Medication adherence reporting scale-5 (MARS-5) was used to assess adherence to antiepileptic drugs. The Oslo social support, Jacob perceived stigma scale, and hospital anxiety and depression scale (HADS) were the instruments used to assess associated factors. Simple and multiple linear regression analysis models were fitted. Then, the adjusted unstandardized beta (β) coefficient at a 95% confidence level was used.

**Results:**

The mean(SD) score of antiepileptic medication adherence was 16.38(±3.76) with 95%CI:(16.03, 16.72). Depressive symptoms (β= -1.35, 95% CI: (-2.04, -0.65)), anxiety symptoms (β=-1.12,95%CI:(-1,79,-0.44), perceived stigma (β= -1.64, 95% CI:-2.16,-1.12), being single (β=-0.67, 95%CI:-1.20,-0.14), presence of seizure per month(β=-2.11,95% CI: (-2.81,-1.41) and antiepileptic drug adverse effect(β=-0.07,95%CI:-0.11,-0.03) were factors associated with anti-epileptic medication adherence.

**Conclusions:**

The results suggest that the mean score of adherence to antiepileptic drugs was poor as compared to other settings. Antiepileptic medication adherence screening tool should be included in the patient's treatment protocol.

## Introduction

Epilepsy is characterized by a recurrent episodes of unprovoked seizures with or without loss of consciousness (1). Globally, around 69 million people were affected. The majority, 90% lives in developing countries, for instance in sub-Saharan African countries (2, 3). Epilepsy in Ethiopia is one of the public health burden. T he prevalence of the disease in the country was reported to be 5.2/1000 inhabitants at risk with the annual incidence of 64 in100,000 inhabitants in rural and community based studies (4,5).

Epilepsy is a treatable disease, and 70 percent of people could be seizure free with optimal anti-epileptic drug (AED) treatment (6). However, many patients with chronic diseases, including epilepsy have difficulty in adhering to their treatment (7). Adherence implies how much the patient behaves in line with medical advice regarding medical usage, modification of lifestyle and follow up visits to the attending health professional (8). Adherence problems are observed in all conditions when the self-administration of medication is required, regardless of the type of disease, its severity, and accessibility to health resources (7).

Medication non adherence might lead to many consequences such as undesirable health outcomes and increase in health-related costs. Negative health outcomes include increase frequency of seizure, medical and psychosocial complications of diseaseand even death (7). Poor adherence to treatment is one of many reasons for pharmacological treatment failure and seizure recurrence (9). The impact of seizure recurrence is multifaceted and extensive in its effect. It has increased the risk of injury, hospitalization and mental health problems, often resulting in anxiety, depression or cognitive impairment. Seizure can also result in stigmatization and social isolation (10). Patients with self-stigma, medication belief, and co-morbid mental illness could result in non-adherent to medication (11,12), which again increases seizure frequency.

Antiepileptic drug non adherence significantly affected by several clinical and demographic factors such as younger age, being male, increasing treatment complexity (13), seizure frequency (14). High costs of antiepileptic drugs, unemployment, more than one antiepileptic drug, side effects of antiepileptic drugs, and non-availability of drugs (8). Substance use behaviors and poor seizure control status have been contributed for the high burden of antiepileptic medication non-adherence (15,16). It is also evidenced that antiepileptic medication non-adherence is associated with a high rate of road accidents, injury and sudden death of patients due to uncontrolled seizure attacks (15,17,18).

Evidences reported that there is a huge treatment gap among people with epilepsy in low and middle income countries (LMIC), ranging from 25 to 100% (19). In Ethiopia, non-adherence to antiepileptic medication ranges from 34.1–65.4% (11,20,21). Though non-adherence to antiepileptic medication has many consequences, it is overlooked by professionals. As a result has received little attention and systematic intervention (7). Limited studies have been conducted to address antiepileptic medication adherence in Africa, including Ethiopia. Measuring adherence is a complex task and there is no gold standard measurement for adherence. Therefore; measuring adherence using different instruments and models could be important to compare different results. In the previous few Ethiopian studies, logistic regression model was used. However, we used linear regression model which might minimize misclassification bias and the study setting was at mental health institutions. Thus, this study will add a great input concerning antiepileptic drug adherence and associated factors among people with epilepsy and it helps to design effective intervention plans and therapeutic techniques to enhance adherence.

## Methods

**Study settings and period**: An institution based cross-sectional study was conducted among people with epilepsy who had follow ups at Amanuel Mental Specialized Hospital, Addis Ababa, Ethiopia in 2019. Amanuel mental specialized hospital is the only specialty mental health hospital in the country since its establishment in 1930, which located in the capital city of Addis Ababa. It gives neuropsychiatric health services through its outpatient and inpatient departments for patients who come from different corners of the country. The hospital has a total of 300 beds and 18 outpatient departments that serve patients with psychiatric disorders, of these three OPDs serve for patients with epilepsy. Many people with epilepsy had follow ups at the outpatient department of the hospital.

**Participants**: Participants of this study were people with epilepsy who had follow ups visit at outpatient department of Amaneul Mental Specialized Hospital, Addis Ababa, Ethiopia. In 2019, an average of 2,398 patients with epilepsy visited the hospital, in one month. Single population mean formula was used to estimate the sample size for antiepileptic drug adherence with the assumption of M (SD), 13.42(±6.53), 1.96 Z (standard normal distribution), 95% CL, α=0.05, and 1.3 margin of error(22). By adding a 10% non-response rate, the estimated sample size was 449. Participants were selected using the systematic sampling technique for the interviews. All patients aged 18 years and above who were diagnosed with epilepsy and provided with treatment in the outpatient neurologic department were included, whereas participants with intellectual disability were excluded.

**Measurement**: Medication adherence reporting scale-5 (MARS-5) was used to measure adherence to anti-epileptic drugs. It is a self-reported instrument containing five items regarding medication adherence. The items were rated on a 5-point Likert scale and the total score ranged between 5 and 25 and a higher score indicates a better medication adherence (23). Though this instrument is short and measures adherence to medication, as per our knowledge, it has not been used yet among patients with epilepsy in Ethiopia. It was difficult to compare the validity and internal consistency with previous studies in Ethiopia. However, we conducted a reliability analysis for the Amharic version of the questionnaire, it was cronbach's α=0.75.

Social support was assessed through the Oslo social support scale. The scale ranged from 3–14 and has three categories the scores 3–8 poor support, 9–11 medium support and 12–14 strong support (24). Hospital Anxiety and Depression Scale (HADS) was used to measure anxiety and depressive symptoms among patients with epilepsy. The tool has 14 items and two subscales, i.e. anxiety subscale (HADS-A) and depression subscale (HADS-D). From 14 items, 7 items were measuring cognitive and emotional aspects of depressive symptoms, and the remaining 7 items assessed anxiety symptoms. Hospital Anxiety and Depression Scale was a reliable and valid instrument in Ethiopia (25). Patients who scored ≥ 8 for both anxiety and depression symptoms were considered as having depression and anxiety symptoms.

Three items Jacoby perceived stigma scale was used to assess the perceived stigma in patients with epilepsy. It was originally developed for assessment of perceived stigma in stroke. Then, it was shown to have good psychometric properties; and was subsequently adapted for epilepsy perceived stigma measurement. Three of the items require a simple “yes/no” response (26).

Liverpool Adverse Events Profile was used to assess AEDs side effectswith in the last one month. It contains 19 items rated on a 4-point Likert scale. The total score ranges from 19 to 76, and the high score indicates more-frequent side effects were experienced (27). Substance use history was assessed by ASSIST, which is a brief screening tool developed by World Health Organization. This tool was used to assess the current and ever psychoactive substance use of the respondents (28). Items on socio-demographic factors (age, sex, ethnicity, religion, marital status, educational status and occupational status) were adapted from a variety of literatures.

**Data collection**: High concerns were given in preparing the data collection instruments for its simplicity to maintain quality data. Before the actual data collection, pretest was done on 5% of the sample in other health institution. Data were collected by three mental health professionals using the Amharic version of the questionnaire for a month. The questionnaire was originally prepared in English, translated into the Amharic version (National Language) and then back translated to English to check its consistency. Training on the aim of the study, interviewing skills, recruitment of participants and the ethical aspects of the data collection was given to data collectors and supervisor.

**Data processing and analysis**: The data were coded and entered into EpiData version 3.1, then exported SPSS version-20 for analysis. Descriptive statistics (frequency, mean, percent and standard deviation) were used to summarize the data. Linear regression assumption tests were fitted before analysis. Then simple linear regression was performed to identify factors associated with AED medication adherence. Variables with a p-value ≤0.05 during simple linear regression were entered into multiple linear regression for further analysis. The multiple coefficient of determination (R^2^) was used for the model fitness. Factors associated with AED adherence were described by unstandardized “β” coefficient with a 95% confidence level, and p-value <0.05 was declared as statistically significant.

**Ethical consideration**: Ethical assurance was obtained from the joint Ethical Review Committee (ERC) of the University of Gondar and Amanuel Mental Specialized Hospital (Ref No: UoG/20/45/8/26/11). Written consent informed was obtained from the participants after explaining the purpose and benefit of the study. The study subjects were assured confidentiality by anonymity of their identification.

## Results

A total of 439 participants took part in the study, with a response rate of 97.8% (Non response were due to refusal). More than half of the study participants (52.6%) were single and 284 (64.7%) were males. More than three-fifths (62.2%) were Orthodox Christian;120 (27.3%) were in the age ranges of 26–33 years;one-third (33.3%) of the participants were Amhara by ethnicity and 182(41.5%) were unemployed. Regarding educational status,129(29.4%) had secondary education (Table 1).

**Table 1:** Socio-demographic variables of patients with epilepsy on follow-ups at Amanuel Mental Specialized Hospital, Ethiopia, 2019 (n=439)

Variables	Categories	Frequency (%)
**Age**	18–25	97 (22.1)
	26–33	120 (27.3)
	34–41	116 (26.4)
	42–49	63 (14.4)
	50 and above	43 (9.8)
**Sex**	Male	284 (64.3)
	Female	155 (35.3)
**Religion**	Orthodox	273 (62.2)
	Muslim	101 (23)
	Protestant	53 (12.1)
	Others*	12 (2.7)
**Ethnicity**	Amhara	146 (33.3)
	Oromo	134 (30.5)
	Tigre	20 (4.6)
	Gurage	116 (26.4)
	Others**	23 (5.2)
**Marital** **status**	Single	231(52.6)
	Married	163 (37.1)
	Divorced/Widowed	45 (10.3)
**Educational** **status**	No formal education	94 (21.4)
	Primary	126 (28.7)
	Secondary	129 (29.4)
	Tertiary and above	90 (20.5)
**Occupational** **status**	Employed	114 (25.9)
	Private business	122 (27.8)
	Unemployed	182 (41.5)
	Others***	21 (4.8)

* Catholic, wakefeta, Hawariyat

** Hadia, wolayta, Gamo, Afar

*** House wife, Student

**Clinical factors for antiepileptic drug non adherence**: The mean (±SD) age for the onset of the illness of the participants' was 19.73(±10.42) years, and more than three-fifths (62%) of patients with epilepsy were ill more than 11 years. Of the respondents, 165(37.6%) had a seizure per month, and 307(69.9%) of the patients took single type of antiepileptic drugs. 158(36%) and 155(35.3%) of the participants had depressive and anxiety symptoms, respectively. A small number, 55(12.5%) of the participants had co-morbid medical illness (Table 2).

**Table 2:** Clinical factors for antiepileptic drug adherence among patients with epilepsy at Amanuel Mental Specialized Hospital, Ethiopia, 2019 (n=439)

Variables	Category	Freq	Percentage/mean
Age of onset of the illness(year)(M±SD)			19.73±10.42
Side effect of AED (M±SD)			36.67±9.47
Duration of illness	≤5 years	75	17
	6–10 years	92	21
	≥11 years	272	62
Presence of seizure per month	No	274	62.4
	Yes	165	37.6
Drug therapy	Monotherapy	307	69.9
	Polytherapy	132	30.1
Depression	Yes	158	36
	No	281	64
Anxiety	Yes	155	35.3
	No	284	64.7
Co-morbid medical diagnosis*	Yes	55	12.5
	No	384	87.5

* Diabetes Mellitus, HIV/AIDS, Hypertension and Congestive Heart Failure

**Psychosocial and substance related characteristics of respondents**: Psychosocial characteristics of the participants showed that 203 (46.2%) had poor social support, and 227 (51.7%) had perceived stigma (Table 3).

**Table 3:** Psychosocial and substance related factors for antiepileptic drug adherence among patients with epilepsy at Amanuel Mental Specialized Hospital, Addis Ababa, Ethiopia, 2019 (n=439)

Variable	Categories	Frequency
**Social support**	Poor	203 (46.2)
	Moderate	120 (27.4)
	Strong	116 (26.4)
**Perceived stigma**	Stigmatized	227 (51.7)
	Non-stigmatized	212 (48.3)
**Current substance use**	Yes	135 (30.8)
	No	304 (69.2)

**Figure 1:**
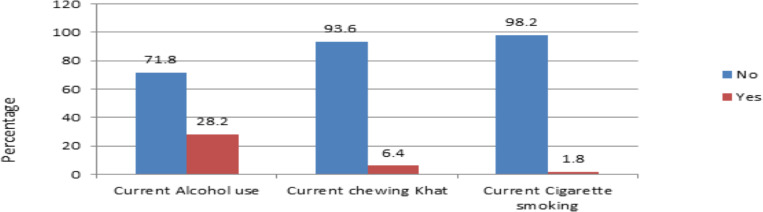
Substance related factors forantiepileptic drug adherence among patients with epilepsy at Amanuel Mental Specialized Hospital, Addis Ababa, Ethiopia, 2019 (n=439).

About 135 (30.8%) of the participants were using substance for non-medical purpose at the moment (Figure 1).

**Adherence to antiepileptic drug**: The mean score of antiepileptic medication adherence was 16.38 (95%CI: 16.03,16.72) with standard deviation of 3.76.

**Factors associated with antiepileptic drug non adherence**: Simple linear regression indicated: educational status, presence of seizure per month, marital status, social support, perceived stigma, depression and anxiety, co-morbid medical conditions, and presence of AED side effect were factors negatively associated with antiepileptic medication adherence with p-value ≤0.05. These factors were entered to multiple linear regressions to control confounding factors. Results of multiple linear regression showed that: perceived stigma, presence of depressive and anxiety symptoms, marital status, presence of seizure per month, and presence of antiepileptic drug adverse effects were negatively associated with antiepileptic drug adherence with p-value ≤0.05 (Table 4).

**Table 4:** Simple and multiple linear regression analysis showing influencing factors for antiepileptic drug adherence among patients with epilepsy attending follow ups at Amanuel mental specialized Hospital, Ethiopia, 2019

Variables		Crude β(95% CI)	Adjusted β(95% CI)
Constant		-	22.22 (20.75,23.69)
Education	No formal education	-1.33(-1.29,-0.48)	-0.02(-0.80,0.75)
	Primary	-0.07(-0.86,0.71)	0.21(-0.49,0.90)
	Secondary	0.46(-0.31,1.24)	0.30(-0.38,0.99)
	Tertiary	Ref	Ref
Presence seizure per month	Yes	-4.81(-5.38,-4.24)	-2.11(-2.81,-1.41)
	No	Ref	Ref
	Single	-0.94(-1.65,-0.24)	-0.67(-1.20,-0.14)
Marital status	Divorced /Widowed	0.42(-0.75,1.59)	0.19(-0.65,1.04)
	Married	Ref	Ref
	Strong social support	Ref	Ref
	Moderate social support	1.87(1.10,2.65)	-0.16(-.81,0.48)
Social support	Poor social support	-3.46(-4.09,-2.83)	-0.14(-0.86,0.59)
	Non-stigmatized	Ref	Ref
Perceived stigma	Stigmatized	-3.45(-4.07,-2.8)	-1.64(-2.16,-1.12)
Depression	No	Ref	Ref
	Yes	-4.51(-5.11,-3.90)	-1.35(-2.04,-0.65)
Anxiety	No	Ref	Ref
	Yes	-4.37(-4.99,-3.76	-1.12(-1.79,-0.44)
Co-morbidity	No	Ref	Ref
	Yes	-1.45(-2.51,-0.39)	-0.46(-1.19,0.27)
Antiepileptic drug adverse effect		-0.22(-0.25,-0.19)	-0.07(-0.11,-0.03)

CI=confidence interval, β= un standardized co-efficient, ref=reference

## Discussion

Adherence to medication remains an important problem in treatment. In this study, as assessed by MARS-5, the mean score of antiepileptic drug adherence was 16.38 (95%CI: 16.03, 16.72). This study showed higher medication adherence score in epileptic patients, as compared to other studies that used the same measurement have reported 13.32 to13.46 mean score of medication adherence (22,29,30). The discrepancy might be due to socio cultural difference, sample size and study design differences. For instance, longitudinal study design was used in Iranian study; whereas cross-sectional study design was used in this study. Because measuring adherence is a complex task, longitudinal measuring of adherence is relatively accurate than one time measurement of adherence. The finding indicated that healthcare providers should emphasize medication adherence among their patients.

Regarding predictor variables, being single was negatively associated with adherence to antiepileptic drugs. The score of adherence was decreased by 0.67 when the participants were single in marital status. This finding was supported by another study (13). It might be related to single participants might not have social assistant in memorizing the time, the dose of the medication. As a study showed (13)47.5% of the participants were forgetting to take their medication. In our finding more than half, (51.9%) of the participants were forgetting to take their medication. Of 51.9% of forgetful participants, about 57.5% were single. The social support including family and friends may improve the patients' confidence on treatment and influences adherence. The other possible reasons might also be related to physical and psychological dysfunction, which all leads to poor controlled epilepsy and the considerable financial impact on health services and influence adherence. Psychological support to address adherence may well be a very cost effective (14), and therefore, this participants may benefit from psychological support. In addition, these single participants might have poor life style management. Participants with poor life style management could not have healthy living in patients with epilepsy, where medication adherence is within life style management (22).

The other predictor variable which affects adherence was the occurrence of seizure episode within a month. It is widely accepted that non-adherence leads to increased seizure frequency; the findings also suggest that uncontrolled seizures paradoxically, contribute to non-adherence. The score of adherence to medication was decreased by 2.11in every unit increase seizure attack per month. This association was supported by other study results (13). This might be related to patient's perception of the treatment and seizure control. Patients may perceive that the treatment is not working if they experience a single seizure attack, and may discontinue the medication or they might take more than the dose prescribed. Non-adherence could be described as reduced or increased amount of a single dose; decreased or increased number of daily doses; incorrect dosing intervals, taking discontinued medication; discontinuing prescribed medication and skipping to take medication (7,31). Higher perceived epilepsy-related stigma was negatively associated with adherence. This finding was supported by other study findings (32). Stigma might lead to poor access to health care and non-adherence or decreased adherence to treatment, ultimately increasing morbidity and mortality.

Epileptic patients with co-morbid depression and anxiety symptom were negatively associated with adherence to their treatment. Depression and anxiety were negatively associated with adherence. Our finding is consistent with findings noted in other study (33,34). This might have many potential explanations. Low mood, sense of hopelessness, and loss of interest could result in low motivation to take medication properly (35). Fatigue, sleep disturbance and loss of concentration could lead to impairment in daily functioning including forgetting medication intake (36). Depression could also lead to lessen proper communication between health care provider and patient which, in turn, could derive non adherence (37). In addition, depressive symptoms could diminish patient expectation of the benefits of the AED treatment and could accentuate pessimism about the downsides of taking medications (36). Patients with anxiety could have low confidence and difficulty in remembering to take AEDs (34). Moreover, Patients with antiepileptic drug side effects had a negative association with adherence. Patients with antiepileptic drug side effect could have concerns about side effects which, in turn, lead to lack of motivation to take the drug. This finding is supported by other study findings (38). It might also be related to higher frequency of side effects among patients taking AEDs.

The main limitation of this study was that the instrument measuring adherence in the survey was not validated in Ethiopia by an expert panel. However, the questionnaires have acceptable psychometric properties to measure adherence. Secondly, severity of the illness or non-responsiveness to treatment might influence adherence, but we haven't assessed these issues.

Anti-epileptic medication adherence was poor among Ethiopian patients with epilepsy in outpatient department. Being single, having one seizure attack per month, high perceived stigma, depression and anxiety symptoms and presence of anti-epileptic drug side-effects were factors associated with adherence problems. It's important that antiepileptic medication non adherence screening tool should be included in the patient treatment protocol. Furthermore, prevention, early screening and intervention of co-morbid mental illness and medication side effects are vital to promote anti-epileptic medication adherence in Ethiopia.
